# Beneficial Effects of Cyclic Ether 2-Butoxytetrahydrofuran from Sea Cucumber *Holothuria scabra* against Aβ Aggregate Toxicity in Transgenic *Caenorhabditis elegans* and Potential Chemical Interaction

**DOI:** 10.3390/molecules26082195

**Published:** 2021-04-11

**Authors:** Taweesak Tangrodchanapong, Nilubon Sornkaew, Laphatrada Yurasakpong, Nakorn Niamnont, Chanin Nantasenamat, Prasert Sobhon, Krai Meemon

**Affiliations:** 1 Department of Anatomy, Faculty of Science, Mahidol University, Bangkok 10400, Thailand; naem400@hotmail.com (T.T.); barbaraormoll@gmail.com (L.Y.); prasertsobhon@gmail.com (P.S.); 2 Department of Chemistry, Faculty of Science, King Mongkut’s University of Technology Thonburi, Bangkok 10140, Thailand; nilubon_jib@hotmail.com (N.S.); nakorn.nia@kmutt.ac.th (N.N.); 3 Center of Data Mining and Biomedical Informatics, Faculty of Medical Technology, Mahidol University, Bangkok 10700, Thailand; chanin.nan@mahidol.edu

**Keywords:** Alzheimer’s disease, amyloid-β, 2-butoxytetrahydrofuran, *C. elegans*, *H. scabra*, sea cucumber

## Abstract

The pathological finding of amyloid-β (Aβ) aggregates is thought to be a leading cause of untreated Alzheimer’s disease (AD). In this study, we isolated 2-butoxytetrahydrofuran (2-BTHF), a small cyclic ether, from *Holothuria scabra* and demonstrated its therapeutic potential against AD through the attenuation of Aβ aggregation in a transgenic *Caenorhabditis elegans* model. Our results revealed that amongst the five *H. scabra* isolated compounds, 2-BTHF was shown to be the most effective in suppressing worm paralysis caused by Aβ toxicity and in expressing strong neuroprotection in CL4176 and CL2355 strains, respectively. An immunoblot analysis showed that CL4176 and CL2006 treated with 2-BTHF showed no effect on the level of Aβ monomers but significantly reduced the toxic oligomeric form and the amount of 1,4-bis(3-carboxy-hydroxy-phenylethenyl)-benzene (X-34)-positive fibril deposits. This concurrently occurred with a reduction of reactive oxygen species (ROS) in the treated CL4176 worms. Mechanistically, heat shock factor 1 (HSF-1) (at residues histidine 63 (HIS63) and glutamine 72 (GLN72)) was shown to be 2-BTHF’s potential target that might contribute to an increased expression of autophagy-related genes required for the breakdown of the Aβ aggregate, thus attenuating its toxicity. In conclusion, 2-BTHF from *H. scabra* could protect *C. elegans* from Aβ toxicity by suppressing its aggregation via an HSF-1-regulated autophagic pathway and has been implicated as a potential drug for AD.

## 1. Introduction

Alzheimer’s disease (AD) is the most common neurodegenerative disorders and was reported in a recent study as the third largest contributor to global, neurological, disability-adjusted life-years [1]. Thus, in order to combat this disorder and improve quality of life, the discovery of an effective treatment for AD is urgently needed. Analytical research indicated that among neurodegenerative diseases related to protein aggregation, AD showed the highest relevance to be curable by natural products (up to 79%) [2]. This is one of the reasons that metabolites from natural organisms—either plants or animals—may contain the derivatives needed to develop therapeutic agents against AD. *Holothuria* (*Metriatyla*) *scabra Jaeger* (*H. scabra*) is one of the most edible sea cucumber species and is widely used as traditional medicine in Asian countries due to its highly diverse natural compounds with nutritional and pharmacological properties [3,4]. Our previous studies demonstrated that its extracts could activate the insulin/insulin-like growth factor 1 (IGF-1) signaling (IIS) pathway for lifespan extension [5]. In that pathway, its downstream targets, transcription factor heat shock factor 1 (HSF-1) and abnormal dauer formation 16 (DAF-16/Forkhead box O (FOXO) homolog), can also play a regulatory role in protecting *Caenorhabditis elegans* from toxic amyloid-β (Aβ) aggregation, a putative etiology of AD [6,7]. Additionally, the extracts could exert a significant effect on reducing α-synuclein aggregation in the *C. elegans* model of Parkinson’s disease (PD) [8]. Regarding this therapeutic potential against the aggregation of α-synuclein, as categorized in a group of proteinopathy-causing diseases including AD by Aβ [9,10], we hypothesized that purified compounds isolated from the *H. scabra* sea cucumber may have similar mitigatory effect against Aβ aggregates via IIS’s transcription factor.

Particularly, Aβ fibril deposition, which is believed to be the main precipitating factor in AD pathology, is derived from disordered peptides, the proteolytic fragments of amyloid precursor protein (APP) [9,11,12]. Furthermore, oligomers, the intermediate form during the aggregation process, are thought to be the most toxic species that cause neurotoxicity and early cognitive impairment in AD [13,14,15]. Thus, the molecules with abilities to inhibit or reduce the level of toxic oligomer and pathological fibril of Aβ may be used as drugs for AD treatment. Previously, positive results from the testing this strategy had been received by treatment with small ether compounds such as cycloalkyl ether, noladin ether, and 12-crown 4 cyclic polyether [16,17,18,19]. Particularly, the latter with a cyclic structure showed in silico and in vitro anti-Aβ aggregation, and it reduced its toxicity in Aβ42-treated SH-SY5Y cells [18,19]. In addition, by having low molecular weight, it was also able to cross the blood–brain barrier (BBB) to specifically interact with Aβs in mouse AD brains [18]. These findings were showed that ether compounds (in particular with a small cyclic structure) had potential against Aβ aggregation and its toxicity. Interestingly, in our current work, many compounds could be extracted and purified from *H. scabra*, and one major compound is 2-butoxytetrahydrofuran (2-BTHF), a small cyclic ether. Taken together, we hypothesized that 2-BTHF and some other compounds isolated from *H. scabra* may inhibit Aβ aggregation and toxicity. Subsequently, we tested this hypothesis by using transgenic *C. elegans* as an in vivo model for AD and in silico technique to predict the potential target of the bioactive compounds.

*C. elegans* is a well-established model for neurodegenerative diseases including AD. As demonstrated by many researchers, multiple strains of transgenic *C. elegans-*expressing human Aβ peptides could be used in vivo for rapid AD drug screening due to its short life span [20]. Their achievement as a testing model can be seen in many reports [21,22,23]. In this study, we applied three strains: the first was CL4176, in which human Aβ_1–42_ is expressed in the worm’s muscle and its Aβ oligomers’ toxicity results2 in rapid worm paralysis [24,25,26]. The second strain, CL2006, contains an Aβ_1–42_ transgene that can be expressed to form oligomers and fibrils of Aβ_3–42_ peptides in the body wall muscle cells [27]. The last strain, CL2355, expresses human Aβ_1–42_ in pan-neuronal cells, resulting in the defect of a neuronal-controlled behavior—chemotaxis [24]. In addition to multiple strains, a high degree of evolutionarily conserved genes and molecular pathways between *C. elegans* and humans, such as genes regulating autophagy [28] needed for Aβ degradation [29] and the main metabolic IIS genes [30], allowed us to study the mechanisms that may mediate the actions of bioactive compounds from *H. scabra* in *C. elegans*.

In this study, we aimed to determine the anti-Aβ therapeutic properties of *H. scabra* isolated compounds by using the *C. elegans* AD model. Among five *H. scabra* compounds, we found that 2-BTHF had the highest effect in protecting the worms from Aβ toxicity. The potential mechanism by which 2-BTHF inhibited Aβ toxicity was mediated through the HSF-1 regulating autophagic pathway needed for Aβ degradation. This was evidenced in the lowering of Aβ aggregates and their derivatives, reactive oxygen species (ROS). 2-BTHF also possessed an in vivo neuroprotective property. Hence, based on these findings, 2-BTHF from *H. scabra* may be considered to be a drug for reducing Aβ aggregate toxicity and curing AD.

## 2. Results

### 2.1. Isolation and Chemical Characterization of H. scabra Compounds

**Figure 1:**
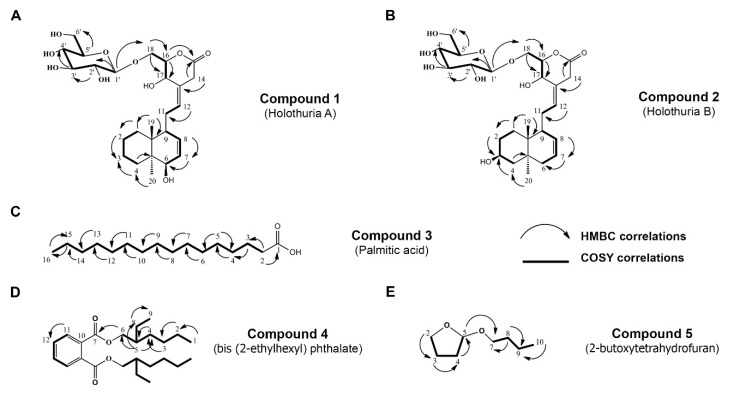
Chemical structure and HMBC and COSY correlations of Compound **1**: 6-hydroxylabda-7(8),12(13)-dien-17-hydroxy-18-*O*-β-d-glucopyranosyl-pyran-15-one (*Holothuria* A) (**A**); Compound **2**: 3-hydroxylabda-7(8),12(13)-dien-17-hydroxy-18-*O*-β-d-glucopyranosyl-pyran-15-one (*Holothuria* B) (**B**); Compound **3**: palmitic acid (**C**); Compound **4**: bis (2-ethylhexyl) phthalate (**D**); and Compound **5**: 2-butoxytetrahydrofuran (2-BTHF) (**E**).

In order to isolate the derivative compounds from *H. scabra* to investigate anti-Aβ therapeutic effect, its ethyl acetate (EtOAc) and butanol (BuOH) extracts were subjected to column chromatography (CC) using a gradient system and then separated by Sephadex LH-20 with an isocratic condition. As a consequent result, five isolated compounds were obtained, and their structures were further elucidated by using ^1^H-NMR, ^13^C-NMR, DEPT 135, electrospray ionization time-of-flight mass spectra (ESI-TOF-MS), HMBC, and COSY correlations as follows: Compound **1**: 6-hydroxylabda-7(8),12(13)-dien-17-hydroxy-18-*O*-β-d-glucopyranosyl-pyran-15-one (*Holothuria* A); Compound **2**: 3-hydroxylabda-7(8),12(13)-dien-17-hydroxy-18-*O*-β-d-glucopyranosyl-pyran-15-one (*Holothuria* B); Compound **3**: palmitic acid (C); Compound **4**: bis (2-ethylhexyl) phthalate (D); and Compound **5**: 2-butoxytetrahydrofuran (2-BTHF) (Figure 1A–E). Chemical information on these isolated compounds are shown in Figures S1–S5 and Tables S1–S5.

### 2.2. Effects of Compounds Isolated from H. scabra on Aβ-Induced Paralysis in Transgenic C. elegans

**Figure 2:**
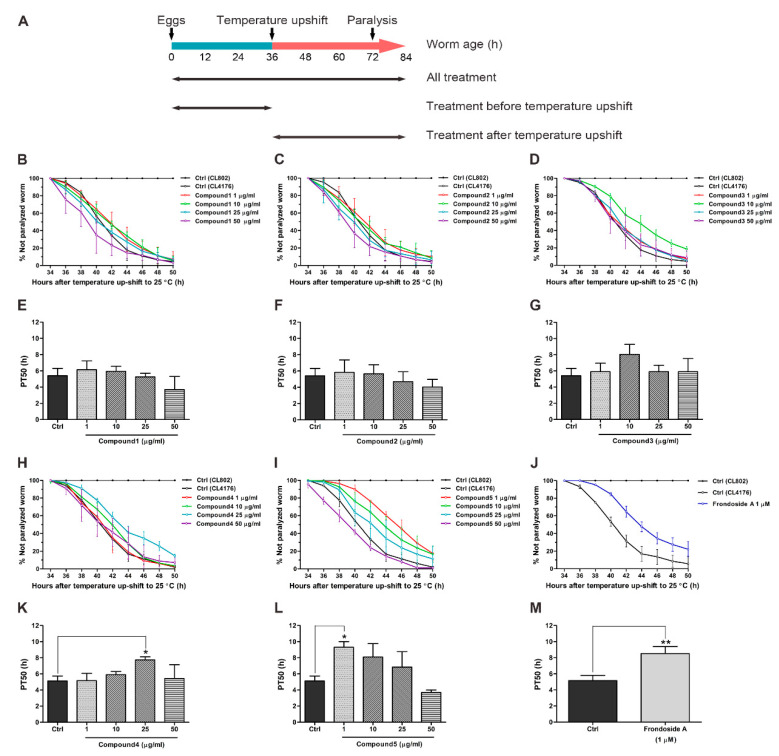
Effect of *Holothuria scabra* isolated compounds on amyloid-β (Aβ)-induced paralysis in *Caenorhabditis elegans* strain CL4176. (**A**) Diagram illustrating the paralysis assays showing the time at which the temperature was increased from 16 to 25 °C in CL4176 and its control stain CL802, as well as the feeding duration of worms with different treatment regiments. (**B**–**D**,**H**–**J**) Time course of paralysis progression caused by Aβ expression in worms treated with either 1% DMSO (Ctrl); different doses of *H. scabra* Compounds **1**, **2**, **3**, **4**, and **5** (1, 10, 25, and 50 µg/mL); or positive control frondoside A at an effective dose 1 µM throughout experimental time. The paralyzed worms were counted at 2 h intervals. Results are represented as percentages ± SD of unparalyzed worms from three independent experiments with at least 100 worms in each experiment. (**E**–**G**,**K**–**M**) The PT50 values were quantified as a mean of time at which 50% worms treated with or without compounds were paralyzed. Error bars indicate SD. * *p* < 0.05 and ** *p* < 0.01 vs. untreated control CL4176 worms.

First, we examined whether isolated compounds from *H. scabra* protected the worms from Aβ-induced toxicity. Five different compounds from *H. scabra* were applied to transgenic CL4176 worms, in which the expression of human Aβ in muscle cells induces paralysis [31]. Similar to a previous work [24], CL4176 worms showed an increased time-dependent paralysis rate during the temperature shift, while CL802 worms (with no expression of Aβ) did not (Figure 2). The results further showed that feeding CL4176 worms with five isolated compounds throughout experimental period (all treatment) caused different levels of anti-paralytic responses. Compounds **1**–**3** had no ability to delay paralysis progression in the treated worms (Figure 2B–D), whereas a significant delay of paralysis was observed compared with untreated control by Compound **4**, and the delay was more notable with Compound **5** treatment (Figure 2H–I). For quantitative analysis, the PT50 value was defined as a mean of the time interval at which 50% of worms were paralyzed after temperature shift. Consistent with % unparalyzed worms, no statistical significances of increased PT50 were found at all concentrations of Compounds **1**–**3** (Figure 2E–G). In contrast, the PT50 was gradually increased in a dose-dependent manner in worms treated with Compound **4** and reached maximum at 25 µg/mL, at which the PT50 was significantly higher than the untreated control, before dropping again at the highest concentration at 50 µg/mL (Figure 2K). In Compound **5** treatment, the worms exhibited a significant increase of PT50 at 1 µg/mL, and then the PT50 was reduced in a dose-dependent manner but still higher than control from 10 to 25 µg/mL and reached the control level at 50 µg/mL (Figure 2L). These data indicated that among five isolated compounds, Compound **5** was the most effective in protecting the worms against Aβ-induced paralysis. Moreover, this protective effect of Compound **5** was superior to frondoside A (an appositive control) with a higher PT50 by approximately 4.3% (Figure 2J,M). However, the ability to delay paralysis by Compound **5** tended to be reduced in other experimental conditions in which worms were fed with the compound either before or after Aβ induction (Figure S6). This suggested that a short duration of treatment with the compounds might not be sufficient to attenuate Aβ toxicity.

### 2.3. 2-BTHF (**5**) Alters Expression Level of Aβ Species in Transgenic C. elegans

**Figure 3:**
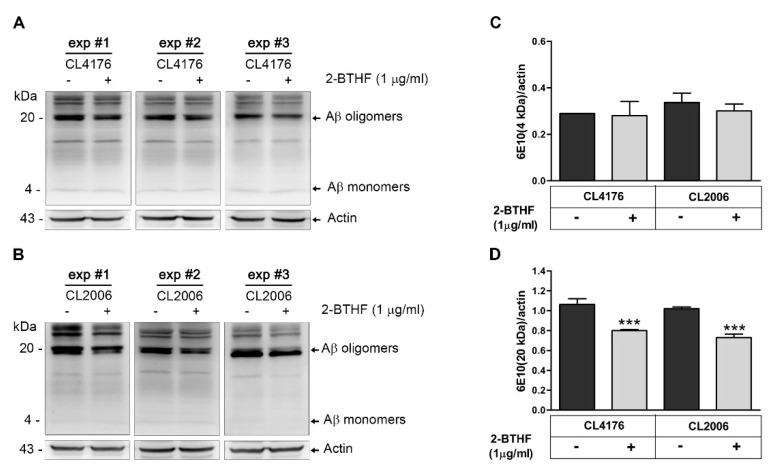
Effect of the bioactive 2-BTHF (**5**) compound on Aβ species in transgenic *C. elegans* strains. (**A**,**B**) Representative Western blot of Aβ species in CL4176 and CL2006 worms treated with or without 2-BTHF (**5**) at 1 µg/mL and detected by anti-Aβ antibody (6E10) or anti-actin. The levels of the Aβ monomer band at around 4 kDa (**C**) and the Aβ oligomer band at 20 kDa (**D**) obtained from untreated and treated worm tissues were quantified by Image J. The arrows indicate actin (43 kDa) or counted Aβ species (4 and 20 kDa). Data are shown as mean ± SD of the indicated band density from three independent experiments (exp) with 1000 worms in each group. *** *p* < 0.001 vs. untreated control.

Because 2-BTHF (Compound **5**) exerted the strongest protective effect against Aβ-induced paralysis in worms, more specific studies on this beneficial effect should be performed. To investigate whether 2-BTHF (**5**) inhibited Aβ oligomerization and possessed an anti-paralytic effect, we carried out Western blotting to quantify the amount of Aβ species in untreated and treated CL4176 worms using antibody against Aβ (6E10). The specific binding of this antibody to human Aβ protein species in transgenic *C. elegans* was previously described [23]. CL2006, which exhibits a progressive reduction of muscle specific motility related to the accumulation of both Aβ_3–42_ fibrils and oligomers, was another strain used for this experiment. The results demonstrated that anti-Aβ monoclonal antibody clone 6E10 (MyBioSource, San Diego, CA, USA) could react with Aβ at several molecular weights in both transgenic CL4176 and CL2006 tissues (Figure 3A,B). Notably, the immunoreactive Aβ oligomer bands at molecular weight of around 20 kDa showed a remarkable decrease in both strains after treatment with 1 µg/mL 2-BTHF (**5**) (Figure 3A,B). Consistent with these observations, the mean densities of Aβ oligomer/actin in both treated CL4176 and CL2006 worms were significantly reduced and lower than the untreated control at approximately 24.5% and 28.4%, respectively (Figure 3D). On the other hand, it was noticed that the density of the Aβ monomer band at molecular weight of around 4 kDa showed non-significant reduction in treated worms compared with vehicle-treated controls (Figure 3C). These findings suggested that 2-BTHF (**5**) primarily suppressed the formation of Aβ oligomers, the main toxic species, hence enabling it to delay Aβ oligomer-induced paralysis in the worms.

### 2.4. Aβ Deposit Is Inhibited by 2-BTHF (**5**) in Transgenic C. elegans

**Figure 4:**
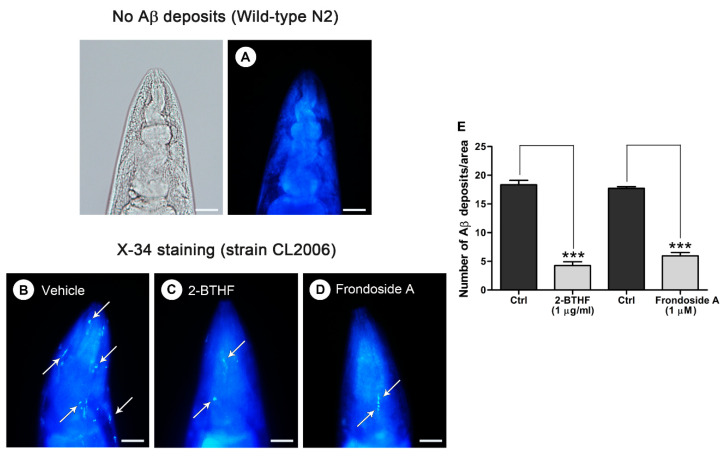
Effect of 2-BTHF (**5**) on Aβ-forming deposits in the CL2006 transgenic *C. elegans* strain. Representative images of *C. elegans* with 1,4-bis(3-carboxy-hydroxy-phenylethenyl)-benzene (X-34) staining in wild-type N2 (**A**) and transgenic strain CL2006 treated with either vehicle (**B**), 2-BTHF (**5**) at 1 µg/mL (**C**), or frondoside A at 1 µM (**D**). White arrows indicate X-34-positive spots of Aβ deposits in the worm’s head, which was separated from the body by a pharyngeal bulb region that is shown in a bright-field image. Scale bars indicate 20 µm. (**E**) Aβ deposits in each group were quantified and expressed as mean number of positive spots ± SEM of Aβ deposits/area in individual worm’s head. Data were obtained in triplicate for each experiment with 23 worms for each analysis (*n* = 69). *** *p* < 0.001 vs. untreated control CL2006.

To further observe whether the inhibitory effect of 2-BTHF (**5**) on Aβ oligomers would reduce degree of amyloidosis, Aβ fibril deposits were quantified in transgenic CL2006 worms. A specific detection of Aβ deposits was carried out by staining with an 1,4-bis(3-carboxy-hydroxy-phenylethenyl)-benzene (X-34) fluorogenic dye that recognizes Aβ aggregates but not oligomers [32]. Our Image-J analysis showed that amyloid-reactive deposits were abundant in the head region of untreated transgenic strain CL2006 but totally absent in the negative control wild-type N2 (Figure 4A,B). On the other hand, a robust reduction of the X-34 positive spots that represent Aβ deposits was observed in CL2006 worms treated with 1 µg/mL 2-BTHF (**5**) (Figure 4C,E; Ctrl, 18.3 ± 0.8, vs. 2-BTHF (**5**), 4.3 ± 0.7; *p* < 0.001). A similar result was obtained from the treatment with frondoside A at 1 µM, which was used as a positive control (Figure 4D,E; Ctrl, 17.7 ± 0.3, vs. frondoside A, 5.9 ± 0.6; *p* < 0.001). These data suggested a strong inhibition of Aβ-oligomer formation and deposition by 2-BTHF (**5**).

### 2.5. Effects of 2-BTHF (**5**) on Autophagy-Regulated Genes and Transcriptional Activities in Insulin-Like Growth Factor Signaling (IIS) Pathway in Transgenic C. elegans

**Figure 5:**
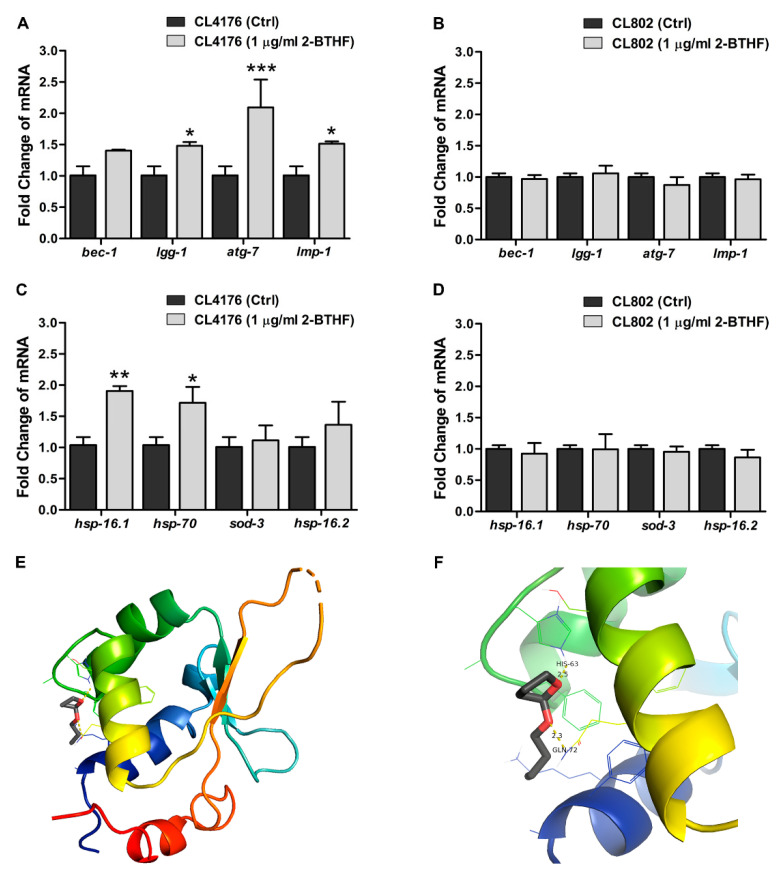
Effects of 2-BTHF (**5**) on the expression levels of genes involved with autophagy, transcriptional activities in IIS pathway in *C. elegans* strains, and its potential target on heat shock factor 1 (HSF-1) molecule. mRNA levels of autophagy-related genes in CL4176 (**A**) or CL802 (**B**) treated with vehicle or 2-BTHF (**5**) at 1 µg/mL. mRNA levels of HSF-1 and dauer formation 16 (DAF-16) target genes in CL4176 (**C**) or CL802 (**D**) treated with or without the compound. All mRNA expression levels in worms were quantified by quantitative RT-PCR at 36 h after temperature increase and normalized with the internal control *act-1*. Data are expressed as mean ± SD from three independent experiments. * *p* < 0.05, ** *p* < 0.01, and *** *p* < 0.001 vs. untreated control CL4176 worms. (**E**) Molecular docking model of 2-BTHF (**5**) binding to HSF-1. (**F**) 2-BTHF (**5**) molecule is bound to the DNA-binding domain (DBD) of HSF-1 at residues HIS63 and GLN72, shown in a magnified docking image.

In many recent studies, the ability to enhance autophagy by nutraceuticals has been required for reducing the paralysis progression caused by Aβ aggregate toxicity in transgenic *C. elegans* worms [33,34,35]. Thus, we investigated whether the protective mechanism of 2-BTHF (**5**) against toxicity by Aβ aggregation might be mediated through this pathway. Quantitative real-time PCR revealed that 2-BTHF (**5**) at 1 µg/mL affected the expression levels of genes involved in several steps of autophagy (Figure 5). 2-BTHF (**5**)-treated CL4176 worms exhibited a moderate upregulation of *bec-1* (encoding the protein that initiates autophagy induction) and significant increases of *lgg-1* (autophagosome elongation), *atg-7* (autophagosome formation), and *lmp-1* (autophagosome-lysosome fusion) mRNA levels, which were higher than untreated controls by approximately 1.5-, 2.1-, and 1.5-folds, respectively, at 36 h after temperature upshift (Figure 5A). In contrast, these mRNA levels were not significantly different between untreated and treated groups when 2-BTHF (**5**) was given to CL802 worms (Figure 5B). These data implied that genes regulating the autophagic pathway were only upregulated by 2-BTHF (**5**) when Aβ aggregation was presented in the worms, specifically leading to its attenuative action on Aβ-induced toxicity.

Moreover, our previous study [5] demonstrated that *H. scabra* extracts could promote the IIS pathway, and the upregulations of its downstream transcription factors HSF-1 and DAF-16 were found to exert protective action against Aβ aggregate proteotoxicity in a transgenic *C. elegans* model [6]. Therefore, to examine the influence of 2-BTHF (**5**) on these transcription factors, we quantified the transcriptional activities of HSF-1 and DAF-16 by analyzing transcriptional expression of their most reliable downstream target genes. The *hsp-16.1* and *hsp-70* are classical readouts for HSF-1, as are *sod-3* and *hsp-16.2* for DAF-16. The results demonstrated that compared with untreated control, there were significant increases of *hsp-16.1* and *hsp-70* by 1.9- and 1.7-folds, respectively, in 2-BTHF (**5**)-treated CL4176 worms (Figure 5C). On the other hand, the mRNA levels of *sod-3* and *hsp-16.2* were not significantly altered between treated and untreated CL4176 worms. These data suggested that the action of 2-BTHF (**5**) in protecting against Aβ aggregate toxicity might be mediated through the stimulation of transcriptional activities of HSF-1 rather than DAF-16. This action was also dependent on the presence of Aβ aggregation, as there was no observable effects in the expression levels of these genes in 2-BTHF (**5**)-treated CL802 worms (Figure 5D). Basically, HSF-1 is comprised of a conserved N-terminal winged helix-turn-helix DNA-binding domain (DBD) followed by a coiled-coil oligomerization domain (HR-A/B). The latter is a variable regulatory domain (RegD) that includes a short heptad repeat (HR-C) and a transactivation domain (TAD) [36]. Molecular docking revealed that 2-BTHF (**5**) was bound to HSF-1 via HIS63 and GLN72, which are located in the DBD with hydrogen bond distances of 2.5 and 2.3 Å, respectively (Figure 5E,F). Such binding orientation was considered acceptable if the root mean square deviation (RMSD) was less than 0.2 Å from three trials. Thus, this study suggested that HSF-1 may be a potential target of the 2-BTHF (**5**) molecule. Taken together, these findings indicated that the protective effect of 2-BTHF (**5**) against Aβ aggregate toxicity resulting in paralysis may be mediated by its activations of the autophagic pathway and HSF-1’s transcriptional activities.

### 2.6. 2-BTHF (**5**) Suppresses ROS Production in Transgenic C. elegans

**Figure 6:**
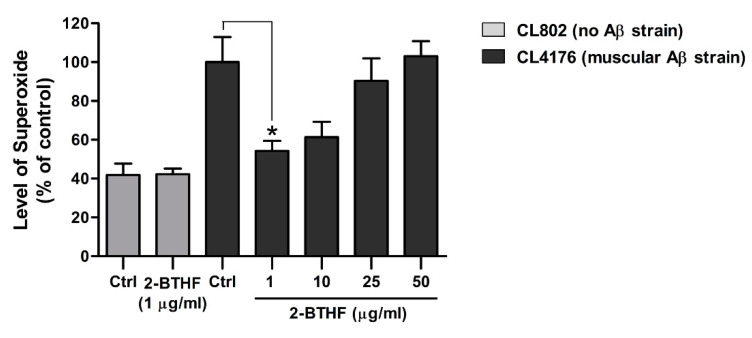
Effect of 2-BTHF (**5**) on reactive oxygen species (ROS) in *C. elegans* strains. The transgenic CL4176 and CL802 *C. elegans* strains were cultured with or without the compounds starting from eggs at 16 °C for 36 h, followed by 25 °C for another 36 h. At the end of experiment, untreated and treated worms were subjected to a 2′,7′-dichlorodihydrofluorescein diacetate (DCF) assay for ROS detection. The ROS levels were expressed as percentage of fluorescence (%DCF) relative to untreated CL4176 control (Ctrl), which was set as 100%. Data were obtained in triplicate for each experiment with 60 worms for each analysis (*n* = 180). Error bars indicate SEM. * *p* < 0.05 vs. untreated CL4176.

Since ROS production from Aβ pro-fibril aggregates has been reported to be a known cause of paralysis in CL4176 worms [37], we evaluated whether the reduction of ROS level by 2-BTHF (**5**) would concurrently occur with the reduction of Aβ oligomers, the main toxic species. As expected, the levels of intracellular ROS in untreated CL4176 worms undergoing temperature shifts were dramatically increased compared with control strain CL802 (Figure 6). Conversely, 2-BTHF (**5**) treatment was able to attenuate the increased level of ROS in a dose-dependent manner, with the lowest dose at 1 µg/mL displaying a significantly reduced ROS level compared to the untreated control (Figure 6: 100.0 ± 13.0% for the control group and 54.2 ± 5.3% for CL4176 worms fed with 1 µg/mL 2-BTHF (**5**)). In contrast to CL4176, the ROS level observed in CL802 did not change between vehicle- and the compound-treated groups at the same experimental condition (Figure 6: 41.9 ± 5.9% for the control group and 42.3 ± 2.9% for CL802 worms fed with 1 µg/mL 2-BTHF (**5**)). These data indicated that the ROS reduction by 2-BTHF (**5**) might specifically be linked to the suppression of the Aβ oligomeric formation that was the cause of paralysis.

### 2.7. 2-BTHF (**5**) Suppresses Aβ Expression in Neurons and Prevents Defect in Chemotaxis Behavior

**Figure 7:**
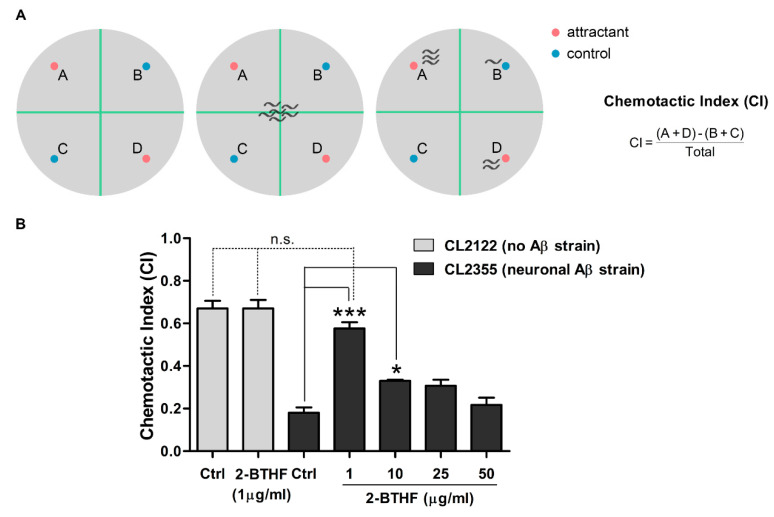
Effect of 2-BTHF (**5**) on chemotaxis behavior in neuronal Aβ-expressing *C. elegans* CL2355 strain. (**A**) Schematic diagram of chemotaxis assay. CL2355 and its control strain CL2122 fed with vehicle or 2-BTHF (**5**) were placed on the center of the assay plate. After incubation for 1 h, migrated worms in each quadrant (A and D with chemical attractant; B and C for control without attractant) were scored and calculated for chemotactic index (CI). (**B**) Representative data are shown as CI ± SD and obtained in triplicate for each experiment with 60 worms for each analysis (*n* = 180) (n.s. *p* > 0.05, * *p* < 0.05, and *** *p* < 0.001).

Chemotaxis is mediated by the activation of several sensory and motor neurons, and it was found to be defective after induced Aβ oligomer formation in the *C. elegans* CL2355 strain [24,38]. This defective behavior could be quantified by a chemotactic index (CI) (Figure 7A) that could be used to evaluate the neuroprotective ability of 2-BTHF (**5**). After their subjection to benzaldehyde attraction and the calculation of their CI, more than 73% of untreated CL2355 worms showed chemotaxis dysfunction, resulting in a significant drop of their CI in comparison with the control CL2122 strain (Ctrl CICL2122, 0.67 ± 0.06, v.s. Ctrl CICL2355, 0.18 ± 0.04; *p* < 0.001) (Figure 7B). Interestingly, treatment with 2-BTHF (**5**) at 1 and 10 µg/mL was able to significantly normalize the CI of CL2355 worms (Figure 7B: 0.18 ± 0.04 for control group and 0.58 ± 0.05 and 0.33 ± 0.01 for CL2355 fed with 1 and 10 µg/mL, respectively). The compound at 1 µg/mL also showed a non-significant different CI value compared with treated or untreated CL2122 worms, suggesting its strong neuroprotective effect (Figure 7B). Even through the effect of 2-BTHF (**5**) in improving the chemotaxis response was certain at low concentrations, its effect was slightly declined when the concentration of the compound was elevated. Additionally, the CI value obtained from treated CL2122 did not change compared with untreated CL2122 (0.67 ± 0.06 for CL2122 fed with vehicle and 0.67 ± 0.07 for CL2122 fed with 1 µg/mL 2-BTHF (**5**)), indicating its non-toxic to animals with healthy neuronal systems. Our results suggested that 2-BTHF (**5**) had high efficacy in attenuating Aβ oligomer formation and neurotoxicity, thus rescuing chemotaxis behavior in AD worms.

## 3. Discussion

In Asian communities, sea cucumbers have long been used as traditional medicine, and a wide range of bioactive compounds have recently been extracted, purified, and used as pharmaceutical and food products [39]. *H. scabra* is abundantly found in Thailand, and our previous study showed that its extracts could rescue *C. elegans* from α-synuclein aggregate-induced PD [8]. Given that this proteinopathy could be the leading cause of neurodegeneration, including the development AD caused by Aβ aggregate [9,10], in the present study, we isolated the compounds from this sea cucumber and investigated their effects in protecting transgenic *C. elegans* from Aβ aggregate toxicity for the first time. Our results revealed that among five isolated compounds, a cyclic ether 2-BTHF (**5**), which is a new chemical structure found in *H. scabra* [40,41,42], exhibited the most effective protection against Aβ-induced paralysis in the treated CL4176 worms. The effect of 2-BTHF (**5**) was dose-dependent, as a low dose of 1 µg/mL displayed the highest protective activity, while the higher doses were less effective in preventing paralysis. In addition to concentration, treatment duration was also another factor of concern, as the treatment of CL4176 worms with 2-BTHF (**5**) before or after the temperature-induced Aβ expression exhibited a minimal anti-paralytic effect compared to continuous treatment (all treatment conditions). Similar results were also observed in CL4176 fed with EGb 761 [24], oleuropein aglycone [43], scorpion venom heat-resistant peptide (SVHRP) [22], and frondoside A [23]. Taken together, these findings suggest to obtain the maximum protective effect in attenuating Aβ-induced toxicity, 2-BTHF (**5**) should be continuously fed to worms.

From previous observation, paralysis, an Aβ-dependent behavior, occurred before detectable Aβ deposition [37]. Thus, 2-BTHF (**5**) may delay paralysis by inhibiting Aβ formation. This notion was supported by immunoblotting, which showed that 2-BTHF (**5**)-treated worms exhibited a significant decrease of the Aβ oligomer band (the main toxic species) at 20 kDa [44,45] in both inducible and constitutive Aβ-expressing strains [21,22,24]. Moreover, a robust reduction of Aβ_3–42_ fibril or plaque deposits that were easily caused by oligomeric assemblies in vitro [46] was also observed in the treated animals. In contrast, the level of the Aβ monomer band at 4 kDa from worms exposed to 2-BTHF (**5**) showed no significant change compared with the untreated control. Many studies implicated that these monomers have physiological roles in modulating normal synaptic plasticity and memory in hippocampal formation [47,48,49], but their assemblies to form larger oligomeric form become toxic [50,51,52]. Taken together, it is likely that the potential action of 2-BTHF (**5**) in delaying paralysis progression in worms is mediated through the reduction of Aβ oligomeric toxic species. Similar results were also obtained in worms treated with frondoside A [23], a positive control used in this study.

The autophagy-lysosomal pathway is an important cellular mechanism needed for eliminating misfolding and abnormally aggregated toxic proteins including Aβ [29]. Therefore, the enhancement of this pathway could ameliorate the amyloid-driven diseases including AD [33,34]. Additionally, in Aβ-expressing worms, the autophagy-lysosomal pathway is essential for a reduction of worm paralysis [35]. These observations were consistent with our results, which showed significant up-regulations of many autophagic genes in CL4176 treated with 2-BTHF (**5**) at 36 h after temperature upshift but not in treated CL802, suggesting that autophagy was only highly activated in Aβ-inducible worms exposed to the compound. This further indicated that the protective action of 2-BTHF (**5**) against paralysis caused by Aβ aggregates in worms might have resulted from autophagic enhancement. It was previously reported by our group that another potential effect of *H. scabra* extracts in anti-PD activity through the lowering of α-synuclein aggregate [8] was mediated via the IIS pathway [5]. This pathway is highly conserved across phyla and plays roles in many biological processes including proteostasis [7]. The IIS pathway mainly functions by activating two downstream transcriptional factors, HSF-1 and DAF-16, which were reported to be the protective mediators against Aβ aggregates and proteotoxicity in worms by affecting targets in the large gene network [6]. Taking a cue from this observation, we therefore hypothesized that 2-BTHF (**5**) mediated protection against Aβ toxicity by influencing these transcription factors. Our findings revealed that 2-BTHF (**5**) enhanced HSF-1’s transcriptional activities rather than DAF-16 in treated CL4176 but not in treated CL802. These findings demonstrated the clear effect of 2-BTHF (**5**) in activating the HSF-1 transcription factor, which might specifically lead to the reduction of Aβ toxicity. In parallel with biochemical responses, molecular docking showed that 2-BTHF (**5**) was bound to the HIS63 and GLN72 residues that are located at the DBD of HSF-1. In a previous work, the interaction between sonneradon A (a compound from *Sonneratia apetala*) and amino acid residues in HSF-1’s DBD were found to promote the conformational change in HSF-1 that resulted in the strengthening of its interaction with related DNA, which thereby enabled the HSF-1 to express related activity in worms [53]. Thus, HIS63 and GLN72 may be key residues that are responsible for modulating the interactions between HSF-1 and DNA. More recently, it was reported that HSF-1 could induce autophagy to improve the survival and proteostasis in *C. elegans* [54]. In supporting this notion, it was demonstrated that in HSF-1-overexpressing worms treated with *bec-1* RNAi, a reduction of PolyQ protein aggregation (i.e., a cause of Huntington’s disease) was not observed compared with treatment with empty vector, thereby suggesting that HSF-1 has a role in the autophagic induction that protected the worms against toxicity of the aggregated protein. By the same token, we suggested that 2-BTHF (**5**) might exerted its anti-Aβ aggregate toxicity in *C. elegans* via the upregulation of autophagy by HSF-1, even though it remains to be determined whether HSF-1 regulates autophagy via direct or indirect mechanisms.

In AD, oxidative stress is associated with Aβ toxicity [55] and believed to be one of the main causes of its pathogenesis [56,57]. Additionally, the increased oxidative stress in CL4176 occurred before detectable Aβ fibril formation, thus suggesting its pre-fibrillar forms as a cause of this stress [37]. In this study, we observed that a significant reduction of ROS simultaneously occurred with the reduction of the Aβ oligomer band in 2-BTHF (**5**)-treated worms. It is likely that the suppressive effect of toxic oligomers by 2-BTHF (**5**) might be further modulated ROS production that did not occur when this compound was given to CL802, a normal control strain. However, the ROS level measured in the normal strain was too low in comparison with CL4176, so the possibility that 2-BTHF (**5**) has direct scavenging effects might not be totally excluded.

2-BTHF (**5**) had beneficial effects not only on the muscle-specific Aβ CL4176 and CL2006 strains but also the pan-neuronal Aβ strain CL2355. In the latter, chemotaxis, a neuronal controlled behavior, was deteriorated by Aβ-induced toxicity [24]. In this respect, we found that 2-BTHF (**5**) could prevent chemotaxis dysfunction because the treated worms showed similar level of response as the normal worms. This indicated a strong ability of 2-BTHF (**5**) in protecting Aβ-induced neurotoxicity in vivo. Similar results in suppressing Aβ oligomers and restoring chemotaxis function were also previously observed in worms treated SVHRP [22] and frondoside A [23]. A number of studies implicated that intraneuronal Aβ oligomers initiated early stage of AD [58,59], which was correlated with early symptoms in AD patients [13,14,15]. Therefore, with their benefits in protecting worms from Aβ oligomer-induced neurotoxicity, 2-BTHF (**5**) could be another alternative drug that should be more studied in advanced preclinical and clinical trials before it can be used to attenuated and/or cure human AD.

Chemically, in this study, five structures of *Holothuria* A (**1**), *Holothuria* B (**2**), palmitic acid (**3**), bis (2-ethylhexyl) phthalate (**4**), and 2-BTHF (**5**) were isolated for the first time from the sea cucumber *H. scabra*. *Holothuria* A (**1**) and B (**2**) are new diterpene glycosides [60,61], while the rest—(**3**) [62], (**4**) [63], and (**5**) [40,41,42]—are known structures. Regarding anti-Aβ’s therapeutic ability, our results strongly indicated that diterpene glycosides including *Holothuria* A (**1**) and B (**2**) had no in vivo anti-Aβ toxic effect in delaying the paralysis of the treated worms. A similar result was also obtained from the treatment with palmitic acid (**3**). These findings demonstrated that those structures may not be mediated for curing Aβ pathology. In contrast, the treatment with bis (2-ethylhexyl) phthalate (**4**) and 2-BTHF (**5**) exhibited a significant effect in protecting the worms from Aβ-induced toxicity. However, the effectiveness of the anti-Aβ toxic action of 2-BTHF (**5**) was higher than bis (2-ethylhexyl) phthalate (**4**). We therefore reasoned that the structure of 2-BTHF (**5**), a small cyclic ether, was more applicable to be further developed as a new drug for treating AD. In addition to 2-BTHF, other ether compounds, particularly with cyclic structures, have shown pharmacological potential in attenuating Aβ aggregation and its toxicity in a multitude of studies [16,17,18,19]. Therefore, this research also increased the evidence that small cyclic ethers should be developed as the therapeutic compounds in halting Aβ pathology and curing AD.

## 4. Materials and Methods

### 4.1. Chemicals and Reagents

Frondoside A (used as a positive compound), benzaldehyde, X-34, and 2′,7′-dichlorodihydrofluorescein diacetate (H2DCF-DA) were purchased from Sigma-Aldrich (St. Louis, MO, USA).

### 4.2. General Procedures for Extraction, Isolation, and Purification

NMR spectra were recorded on a Bruker AVANCE 400 FT-NMR spectrometer (Zurish, Switzerland) operating at 400 MHz for ^1^H-NMR and 100 MHz for ^13^C-NMR, using tetramethyl-silane (TMS) as an internal standard. High-resolution ESI-TOF-MS were measured with a Bruker micrOTOF-QII mass spectrometer (Billerica, MA, USA). CC was carried out using Merck silica gel 60 (particle size less than 0.063 mm), reversed-phase RP C-18 (40–63 µm, E. Merck, Darmstadt, Germany), or Pharmacia Sephadex LH-20. For thin layer chromatography (TLC), Merck precoated silica gel 60 F_254_ plates. For RP-TLC, RP-18 F_254_ precoated on an aluminum plate (E. Merck, Darmstadt, Germany) was used. Spots on a TLC plate were detected under UV light and sprayed with an anisaldehyde-H_2_SO_4_ reagent followed by heating.

### 4.3. H. scabra Preparation for Extraction

Specimens of *Holothuria* (*Metriatyla*) *scabra Jaeger* (*H. scabra*) were collected and identified by the Coastal Fisheries Research and Development Center, Prachuap Khiri Khan, Thailand. The animals were thoroughly cleaned by washing with filtered sea water and anesthetized by freezing them on ice. After that, their body walls were separated, cut into small pieces, and stored at −80 °C. These procedures were ethically conducted under Mahidol University-Institute Animal Care and Use Committee (MU-IACUC; MUSC60-049-399).

### 4.4. H. scabra Extraction and Isolation of Compounds

Small freeze-dried pieces of *H. Scabra* were extracted with ethanol (EtOH) under low pressure to yield the EtOH extract (119.07 g). The EtOH extract was then successively partitioned with EtOAc and BuOH to obtain EtOAc and BuOH extracts, respectively. The EtOAc extract (2.8 g) was fractionated by CC using Sephadex LH-20 with 100% methanol (MeOH) condition. After examining the elutes by TLC, 3 combined fractions (EA1–EA3) were obtained. Fraction EA1 was subjected to CC using *n*-hexane-EtOAc (80:20) to afford Compound **3** (20.8 mg) as a white powder. Fraction EA2 was subjected to CC RP-18 using MeOH-H_2_O (80:20) to afford Compound **1** (20.2 mg) and Compound **2** (12.8 mg). These two obtained compounds were amorphous solids. The BuOH extract (3.5 g) was isolated by CC using a gradient solvent system of *n*-hexane-EtOAc—(80:20), (70:30), and (50:50) with increasing amounts of a more polar solvent of the eluates then examined by TLC. These gave 4 combined fractions (Bu1–Bu4). Fraction Bu2 was chromatographed on silica gel by using CH_2_Cl_2_-MeOH (100:1) to afford Subfractions 1–3. Subfraction 3 was subjected to further CC using *n*-hexane-EtOAc (80:20) to afford Compound **4** (30.20 mg) as a colorless oil. Fraction Bu3 was subjected to CC on silica gel using CH_2_Cl_2_-MeOH (100:1) to afford Subfractions 1–3. Subfraction 2 was further re-chromatographed using *n*-hexane-EtOAc (80:20) to afford Compound **5** (10.30 mg) as an amorphous solid. In these methods, EtOH (ACS), EtOAc (Certified ACS), BuOH (Certified ACS), MeOH (Certified ACS), CH_2_CL_2_ (ACS), and n-hexane (Certified ACS) were obtained from Fisher Scientific, Göteborg, Sweden. Sephadex LH-20 was obtained from GE Healthcare Bio-Sciences AB, Uppsala, Sweden.

### 4.5. C. elegans Strains, Maintenance, and Synchronization

All *C. elegans* strains used in this study were obtained from *Caenorhabditis* Genetics Center (University of Minnesota, Minneapolis, MN, USA) as follows: wild-type N2 (Bristol), transgenic strains CL4176 (Pmyo-3::SP::Aβ_1–42_::long 3’ UTR), CL802 (Pmyo-3; control strain for CL4176), CL2006 (Punc-54::SP::Aβ_1–42_), CL2355 (Psnb-1::SP::Aβ_1–42_::long 3’ UTR + Pmtl-2::GFP), and CL2122 (Punc-54 + Pmtl-2::GFP; control strain for CL2355). In the transgenic *C. elegans* CL4176 and CL2355 strains, the expression of the human Aβ_1–42_ protein was induced by a temperature upshifting from 16 to 25 °C in muscle cells and pan-neuronal cells, respectively. Meanwhile, the CL2006 strain produced body-wall muscle-specific Aβ_3–42_ protein in a constitutive manner. These worm strains were maintained by feeding with the *Escherichia coli* strain OP50, a food source, on a solid nematode growth medium (NGM) at 16 °C—except for CL2006 and wild-type N2, which were maintained at 20 °C. To prepare age-synchronized nematodes, worms were transferred to fresh NGM plates upon reaching maturity at 3 days of age and allowed to lay eggs overnight. The isolated synchronized eggs were then cultured with or without compounds on fresh NGM plates at either 16 or 20 °C. *C. elegans* manipulation was ethically performed under the guidelines of MU-IACUC (MUSC60-051-401).

### 4.6. Dosage Information to C. elegans

All isolated compounds were dissolved in dimethyl sulfoxide (DMSO, VWR International S.A.S., Fontenay-sous-Bois, France) as stock solutions that were stored at –20 °C. Stock solution in DMSO were diluted by directly mixing with *E. coli* strain OP50 to obtain concentrations of the compounds at 1, 10, 25, and 50 µg/mL (with the final concentration of DMSO at 1%) and delivered to synchronized eggs of *C. elegans*. Then, 1% DMSO mixed with *E. coli* strain OP50 was used for the untreated control. The hatched larvae were then exposed and took up the compounds, mainly by the oral route and probably also through the worms’ cuticle [64]. The treated and untreated worms showed no toxic effect, as none incurred death, and the worms underwent normal hatching rates and developing stages.

### 4.7. Paralysis Assay

The synchronized eggs of CL4176 and CL802 (not Aβ-expressing and used as control strain) were placed on fresh NGM plates containing the compounds at 1, 10, 25, and 50 µg/mL and kept at 16 °C. The negative control worms were placed on fresh NGM plates without the compounds, while the positive control worms were placed on fresh NGM plates containing 1 µM frondoside A [23]. After 36 h, when the worms reached the third-stage larvae (L3), the temperature was increased to 25 °C to induce muscle-specific Aβ expression. An experimental profile of paralysis assay was designed in 3 patterns (Figure 1A), as previously described [23]: pattern 1 (all treatment), pattern 2 (treatment before temperature upshift), and pattern 3 (treatment after temperature upshift). Worms that showed body rigidity with no movement or only the movement of their heads after being gently touched with a platinum loop were classified as being paralyzed. Paralysis was scored at 2 h intervals until the last worm became paralyzed. The experiments were repeated three times with at least 100 worms in each trial.

### 4.8. Western Blot of Aβ Species

The Aβ species in the transgenic *C. elegans* strains were identified by immunoblotting following the standard Western blotting protocol [21]. CL4176 worms were cultured with either vehicle or the compounds at 16 °C for 36 h and continued to grow at 25 °C for another 36 h. For the CL2006 strain, the worms untreated or treated with the compounds were kept and maintained at 20 °C for 96 h. At the end of experiment, the worms were collected and washed with ddH_2_O three times. The washed worms were then boiled in lysis buffer containing 62 mM Tris-HCl pH 6.8, 2% SDS (*w*/*v*), 10% glycerol (*v*/*v*), 4% β-mercaptoethanol (*v*/*v*) and a protease inhibitor cocktail (1X, Sigma-Aldrich, St. Louis, MO, USA) at 105 °C for 10 min. After cooling, the samples were centrifuged at 14,000 *g* for 5 min. Equal amounts of total proteins (30 µg), as measured by using an RC DC Protein Assay Kit (Biorad, Hercules, CA, USA), were boiled prior to electrophoresis for 5 min in denaturation buffer (62 mM Tris-HCl pH 6.8, 2% SDS (*w*/*v*), 10% glycerol (*v*/*v*), 4% β-mercaptoethanol (*v*/*v*), and 0.0005% bromophenol blue (*w*/*v*)). The components of the lysis and denaturation buffer—Tris, SDS, glycerol, β-mercaptoethanol, and bromophenol blue—were purchased from Vivantis Technologies Sdn. Bhd. (Selangor Darul Ehsan, Malaysia), Merck KGaA (Darmstadt, Germany), VWR International BVBA (Leuven, Belgium), AppliChem GmbH (Darmstadt, Germany), and Sigma-Aldrich (St. Louis, MO, USA), respectively. After cooling with ice, protein samples were loaded on SDS BIS-Tris gel and run at 140 V in running buffer. The gels were transferred to 0.45 µm polyvinylidene fluoride (PVDF; GE Healthcare, Garching, Germany) membranes using a transferring buffer with 20% methanol. Blots were blocked in TBS-Tween 5% bovine serum albumin (BSA) (100 mM Tris pH 7.5, 150 mM NaCl, and 0.1% Tween-20 (*v*/*v*)). The blocking solution comprising Tris, NaCl, Tween-20, and BSA was obtained from Vivantis Technologies Sdn. Bhd. (Selangor Darul Ehsan, Malaysia), Thermo Fisher Scientific (Waltham, MA, USA), Vivantis Technologies Sdn. Bhd. (Selangor Darul Ehsan, Malaysia), and Capricorn scientific GmbH (Hessen, Germany), respectively. Amyloid protein species were detected with mouse anti-human Aβ1-17 monoclonal antibody clone 6E10 (1:500, MyBioSource, San Diego, CA, USA). Actin was detected by using a mouse anti-α-smooth muscle actin antibody, monoclonal clone 1A4, that was purified from a hybridoma cell culture (1:1000, Sigma-Aldrich, St. Louis, MO, USA). Goat anti-mouse lgG-peroxidase conjugated H+L (1:5000, Sigma-Aldrich, St. Louis, MO, USA) was used as the secondary antibody. Positive bands were developed with the enhanced chemiluminescence (ECL, Thermo Fisher Scientific, Waltham, MA, USA) method and captured by chemiluminescent gel document (Alliance Q9 mini). The mean densities of Aβ signals were analyzed using the Image-J software (National Institutes of Health, NIH, Bethesda, MD, USA). The representative data were from triplicates with approximately 1000 worms in each group (*n*: ~3000).

### 4.9. Fluorescence Staining of Aβ Deposits

Synchronized CL2006 worms cultured with either a vehicle or the compounds were continuously maintained at 20 °C for 120 h. Then, the individual worms were fixed in 4% paraformaldehyde/phosphate buffered saline (PBS), pH 7.4, at 4 °C for 24 h. The fixed worms were stained with X-34 (Sigma-Aldrich, St. Louis, MO, USA), a specific dye for staining Aβ aggregates, in 10 mM Tris pH 8.0 for 4 h at room temperature [32]. The samples were then de-stained, mounted on slides for microscopy, and observed with a fluorescence microscope (Olympus BX53; Olympus Corp., Tokyo, Japan). Fluorescence images were acquired at the same exposure parameters by using a 40x objective of microscope with a CCD camera. Amyloid deposits identified as X-34 positive spots in the worm’s head region were scored and represented as Aβ deposits/area. Each experiment was carried out on 23 worms for each group and independently repeated three times. Wild-type N2 worms, used as negative controls, were also examined using the same procedure.

### 4.10. Gene Expression Analysis by Quantitative RT-PCR

After the treated CL4176 and CL802 worms were transferred from 16 to 25 °C for 36 h, the worms were collected in an M9 buffer for RNA extraction using an RNeasy mini kit (QIAGEN, Hilden, Germany) following the manufacturer’s protocol. The RNA levels were measured by nanodrop (NanoDrop™ 2000/2000c spectrophotometer, Thermo Scientific), and their purity was evaluated by UV absorbance (260/280 ratio). Then, cDNA was synthesized using iScript™ Reverse Transcription Supermix R (Bio-Rad, Hercules, CA, USA). Quantitative real-time PCR was performed in a 10 µL mixture solution of SsoFast™ EvaGreen^®^ Supermix with Low ROX (Bio-Rad, Hercules, CA, USA), nuclease-free water, specific primers, and the cDNA. The primer sequences used in this study are listed in Table 1. PCR reactions were run in a CFX96 Touch Real-time PCR machine (Bio-Rad, Hercules, CA, USA) under the following conditions: real-time PCR was started with 1 cycle of 95 °C for 30 s, followed by 44 cycles at 95 °C for 5 s, and 60 °C for 30 s, and then 75 °C for 30 s to stop the reaction. The expressing Cq values were calculated with equation 2^-ΔΔCq^ for evaluating the fold change in the expression of each gene. The expression levels of mRNA were normalized using the internal control *actin-1 (act-1)*. Data were obtained from three independent experiments with approximately 1000 worms in each group (*n*: ~3000).

### 4.11. Molecular Docking

The three-dimensional molecular structure of 2-BTHF (**5**) was obtained from PubChem (PubChem CID: 2724555). The crystal structure of HSF-1 was obtained from the Protein Data Bank (PDB ID 5d5u) as previously described [53]. The molecular docking of 2-BTHF (**5**) to HSF-1 was carried out using AutoDock4, whereby initial ligand and protein structure preparations (e.g., charge assignments) were carried out using the AutoDockTools version 1.5.6 program. Rendered images of the protein–ligand interaction of the docked complexes were produced using PyMOL version 2.4.1 (Schrödinger, Inc., New York, NY, USA).

### 4.12. Measurement of Reactive Oxygen Species (ROS)

The transgenic *C. elegans* CL4176 and CL802 strains were cultured with or without the compounds starting from eggs at 16 °C for 36 h followed by 25 °C for another 36 h. At the end of experimental period, intracellular ROS in the harvested worms were measured by using H2DCF-DA following the previously described protocol [65]. Briefly, after washing with PBS and 1% Tween 20 (PBST), the worms were sonicated to break up their outer cuticle. Each sample was equally pipetted into three different wells of black 96-well plates (Thermo Fisher Scientific, Waltham, MA, USA) and incubated with a 50 µM H2DCFDA solution (final concentration in PBS) for 30 min at 37 °C. Fluorescent intensity was immediately measured using a Tecan Spark 10M microplate fluorescence reader with the excitation at 485 nm and the emission at 530 nm. Triplicate experiments were carried out for each group of worms, with 60 worms in each trial.

### 4.13. Chemotaxis Assay

Chemotaxis assays were performed following the previously described protocol [66]. Synchronized CL2355 and its control CL2122 worms were cultured with or without the compounds, starting from eggs. The cultured temperature was first set at 16 °C for 36 h and then increased up to 25 °C to induce Aβ_1–42_ expression in the worm’s pan-neuronal cells. At 36 h later, the worms were harvested and assayed in 100 mm plates containing a 25 mM phosphate buffer, pH 6.0, 1 mM MgSO_4_ (May and Baker Ltd., Dagenham, UK), 1 mM CaCl_2_ (Sigma-Aldrich, St. Louis, MO, USA), and 1.9% agar (Oxoid Ltd., Basingstoke, Hants, UK). The assay plate was equally divided into four quadrants with two tests and two controls, as illustrated in Figure 7A. In test areas, 1 µL of attractant (0.1% benzaldehyde, Sigma-Aldrich, St. Louis, MO, USA in 100% ethanol) and 1 µL of 0.25 M sodium azide (Merck Schuchardt, Darmstadt, Germany) were added to the labelled points A and D. On the opposite side of the attractant spots, 1 µL of 100% ethanol and 1 µL of sodium azide were dropped at the control points B and C. After that, the collected worms were immediately placed at the center of the assay plate and incubated at 25 °C for 1 h. The numbers of worms in each quadrant were scored, and the CI was calculated. The CI value was defined as the number of worms at attractant points (A and D) minus the number of worms at control points (B and C) divided by total number of worms as illustrated in Figure 7A. Each assay was done in triplicate using 60 worms in each trial.

### 4.14. Statistical Analyses

The differences between control and compound-treated groups were statistically compared by one-way ANOVA analysis of variance following the Tukey–Kramer test for multiple comparison results or paired *t*-test for two groups. All data analyses were produced by GraphPad Prism version 5.0 (GraphPad Software, San Diego, CA, USA). A *p* value < 0.05 was considered statistically significant.

## 5. Conclusions

In summary, we isolated and purified five compounds from *H. scabra*, and one of these, a cyclic ether 2-BTHF (**5**), exhibited the highest efficacy in alleviating Aβ oligomer-induced toxicity resulted in paralysis inhibition and strong neuroprotective effect in *C. elegans*. Our findings demonstrated that 2-BTHF (**5**) reduced the level of Aβ oligomers, thus possibly halting the formation and deposits of larger Aβ fibrils. In contrast, the compound did not affect the level of monomeric form, which plays physiological roles. 2-BTHF (**5**) also caused the concurrent reduction of ROS-induced oxidative stress in the treated worms. Parallel bioinformatic and in silico studies demonstrated that HSF-1 transcription factor was a potential target of 2-BTHF (**5**), and they together stimulated the upregulation of autophagic genes including *lgg-1*, *atg-7*, and *lmp-1*, whose encoding proteins control the multi-step autophagic process required for breaking down Aβ aggregates and preventing its toxicity. However, more systematic studies on 2-BTHF (**5**) are needed regarding its effects, toxicity, and ability to cross the BBB in higher mammalian animal models before preclinical and clinical trials can begin.

## Figures and Tables

**Table 1:** Oligonucleotide primer sequences for genes used in this study.

Gene	Forward 5’→3’	Reverse 5’→3’
*bec-1*	AGATCTCAAAGCTGCGTGTG	AAAAGGCAGAATTCCAGCAGA
*lgg-1*	AATGGAAACCCAAAGCCCCT	AGGGGAGAAGAGCAACTTCG
*atg-7*	TCTGCAGGATGGATGGTTCG	CTCGGCAAGGTCCATGTGTA
*lmp-1*	CTCGCACCAACGAAGTTGTC	GTATCCGACGAGCACAACCA
*hsp-16.1*	GCAGAGGCTCTCCATCTGAA	GCTTGAACTGCGAGACATTG
*hsp-70*	GCCGGTTGAAAAGGCACTTC	CGAGTTGATCCCCCAACCAA
*sod-3*	GCACACTCTCCCAGATCTCC	TCGAAACAGCCTCGTGAAGT
*hsp-16.2*	GTCACTTTACCACTATTTCCGT	CAATCTCAGAAGACTCAGATGG
*act-1*	ATCGTCACCACCAGCTTTCT	CACACCCGCAAATGAGTGAA

## Data Availability

The data supporting the conclusion in this study are available on request from the corresponding author.

## Supplementary Materials

The following are available online. Figure S1: Chemical analyses of Compound **1** (*Holothuria* A). Figure S2: Chemical analyses of Compound **2** (*Holothuria* B). Figure S3: Chemical analyses of Compound **3** (palmitic acid). Figure S4: Chemical analyses of Compound **4** (Bis (2-ethylhexyl) phthalate). Figure S5: Chemical analyses of Compound **5** (2-BTHF). Figure S6: Effects of Compound **5** (2-BTHF) on Aβ-induced paralysis of *C. elegans* strain CL4176 in treatment before or after temperature up-shifting. Table S1: ^1^H-, ^13^C-NMR and HMBC data of Compound **1** (*Holothuria* A) in CD_3_OD. Table S2: ^1^H-, ^13^C-NMR and HMBC data of Compound **2** (*Holothuria* B) in CD_3_OD. Table S3: ^1^H-, ^13^C-NMR and HMBC data of Compound **3** (palmitic acid) in CDCl_3_. Table S4: ^1^H-, ^13^C-NMR and HMBC data of Compound **4** (Bis (2-ethylhexyl) phthalate) in CDCl_3_. Table S5: ^1^H-, ^13^C-NMR and HMBC data of Compound **5** (2-BTHF) in CDCl_3_.

## Sample Availability

Samples of the compounds are not available from the authors.

## References

[1] GBD 2016 Neurology Collaborators (2019). Global, regional, and national burden of neurological disorders, 1990–2016: A systematic analysis for the Global Burden of Disease Study 2016. Lancet Neurol..

[2] Joyner P.M., Cichewicz R.H. (2011). Bringing natural products into the fold—Exploring the therapeutic lead potential of secondary metabolites for the treatment of protein-misfolding-related neurodegenerative diseases. Nat. Prod. Rep..

[3] Caulier G., Flammang P., Rakotoarisoa P., Gerbaux P., Demeyer M., Eeckhaut I. (2013). Preservation of the bioactive saponins of *Holothuria scabra* through the processing of trepang. Cah. Biol. Mar..

[4] Pangestuti R., Arifin Z. (2018). Medicinal and health benefit effects of functional sea cucumbers. J. Tradit. Complement. Med..

[5] Jattujan P., Chalorak P., Siangcham T., Sangpairoj K., Nobsathian S., Poomtong T., Sobhon P., Meemon K. (2018). *Holothuria scabra* extracts possess anti-oxidant activity and promote stress resistance and lifespan extension in *Caenorhabditis elegans*. Exp. Gerontol..

[6] Cohen E., Bieschke J., Perciavalle R.M., Kelly J.W., Dillin A. (2006). Opposing activities protect against age-onset proteotoxicity. Science.

[7] Cohen E., Dillin A. (2008). The insulin paradox: Aging, proteotoxicity and neurodegeneration. Nat. Rev. Neurosci..

[8] Chalorak P., Jattujan P., Nobsathian S., Poomtong T., Sobhon P., Meemon K. (2018). *Holothuria scabra* extracts exhibit anti-Parkinson potential in *C. elegans*: A model for anti-Parkinson testing. Nutr. Neurosci..

[9] LaFerla F.M., Green K.N., Oddo S. (2007). Intracellular amyloid-beta in Alzheimer’s disease. Nat. Rev. Neurosci..

[10] Irwin D.J., Lee V.M., Trojanowski J.Q. (2013). Parkinson’s disease dementia: Convergence of α-synuclein, tau and amyloid-β pathologies. Nat. Rev. Neurosci..

[11] Haass C., Selkoe D.J. (2007). Soluble protein oligomers in neurodegeneration: Lessons from the Alzheimer’s amyloid β-peptide. Nat. Rev. Mol. Cell Biol..

[12] Benilova I., Karran E., De Strooper B. (2012). The toxic Aβ oligomer and Alzheimer’s disease: An emperor in need of clothes. Nat. Neurosci..

[13] Kayed R., Head E., Thompson J.L., McIntire T.M., Milton S.C., Cotman C.W., Glabe C.G. (2003). Common structure of soluble amyloid oligomers implies common mechanism of pathogenesis. Science.

[14] Nimmrich V., Ebert U. (2009). Is Alzheimer’s disease a result of presynaptic failure? Synaptic dysfunctions induced by oligomeric beta-amyloid. Rev. Neurosci..

[15] Taylor M., Moore S., Mayes J., Parkin E., Beeg M., Canovi M., Gobbi M., Mann D.M., Allsop D. (2010). Development of a proteolytically stable retro-inverso peptide inhibitor of beta-amyloid oligomerization as a potential novel treatment for Alzheimer’s disease. Biochemistry.

[16] Karlstrom S., Soderman P., Swahn B.M., Rakos L., Ohberg L. (2015). Cycloalkyl Ether Compounds and Their Use as Bace Inhibitors. U.S. Patent.

[17] Milton N.G. (2002). Anandamide and noladin ether prevent neurotoxicity of the human amyloid-beta peptide. Neurosci. Lett..

[18] Tian Y., Zhang X., Li Y., Shoup T.M., Teng X., Elmaleh D.R., Moore A., Ran C. (2014). Crown ethers attenuate aggregation of amyloid beta of Alzheimer’s disease. Chem. Commun..

[19] Agrawal N., Skelton A. (2016). 12-crown-4 ether disrupts the Patient Brain-derived Amyloid-βeta Fibril Trimer: Insight from All-atom Molecular Dynamics Simulations. ACS Chem. Neurosci..

[20] Lublin A.L., Link C.D. (2013). Alzheimer’s disease drug discovery: In vivo screening using *Caenorhabditis elegans* as a model for beta-amyloid peptide-induced toxicity. Drug Discov. Today Technol..

[21] Sangha J.S., Sun X., Wally O.S.D., Zhang K., Ji X., Wang Z., Wang Y., Zidichouski J., Prithiviraj B., Zhang J. (2012). Liuwei Dihuang (LWDH), a traditional Chinese medicinal formula, protects against β-amyloid toxicity in transgenic *Caenorhabditis elegans*. PLoS ONE.

[22] Zhang X.-G., Wang X., Zhou T.-T., Wu X.-F., Peng Y., Zhang W.-Q., Li S., Zhao J. (2016). Scorpion Venom Heat-Resistant Peptide Protects Transgenic *Caenorhabditis elegans* from β-Amyloid Toxicity. Front. Pharmacol..

[23] Tangrodchanapong T., Sobhon P., Meemon K. (2020). Frondoside A Attenuates Amyloid-β Proteotoxicity in Transgenic *Caenorhabditis elegans* by Suppressing Its Formation. Front. Pharmacol..

[24] Wu Y., Wu Z., Butko P., Christen Y., Lambert M.P., Klein W.L., Link C.D., Luo Y. (2006). Amyloid-β-induced pathological behaviors are suppressed by Ginkgo biloba extract EGb 761 and ginkgolides in transgenic *Caenorhabditis elegans*. J. Neurosci..

[25] Diomede L., Cassata G., Fiordaliso F., Salio M., Ami D., Natalello A., Doglia S.M., De Luigi A., Salmona M. (2010). Tetracycline and its analogues protect *Caenorhabditis elegans* from β amyloid-induced toxicity by targeting oligomers. Neurobiol. Dis..

[26] Wu Y., Cao Z., Klein W.L., Luo Y. (2010). Heat shock treatment reduces beta amyloid toxicity in vivo by diminishing oligomers. Neurobiol. Aging.

[27] McColl G., Roberts B.R., Gunn A.P., Perez K.A., Tew D.J., Masters C.L., Barnham K.J., Cherny R.A., Bush A.I. (2009). The *Caenorhabditis elegans* Aβ1–42 model of Alzheimer disease predominantly expresses Aβ3–42. J. Biol. Chem..

[28] Chen Y., Scarcelli V., Legouis R. (2017). Approaches for Studying Autophagy in *Caenorhabditis elegans*. Cells.

[29] Tarasoff-Conway J.M., Carare R.O., Osorio R.S., Glodzik L., Butler T., Fieremans E., Axel L., Rusinek H., Nicholson C., Zlokovic B.V. (2015). Clearance systems in the brain—implications for Alzheimer disease. Nat. Rev. Neurol..

[30] Yen C.A., Curran S.P. (2016). Gene-diet interactions and aging in *C. elegans*. Exp. Gerontol..

[31] Link C.D., Taft A., Kapulkin V., Duke K., Kim S., Fei Q., Wood D.E., Sahagan B.G. (2003). Gene expression analysis in a transgenic *Caenorhabditis elegans* Alzheimer’s disease model. Neurobiol. Aging.

[32] Link C.D., Johnson C.J., Fonte V., Paupard M., Hall D.H., Styren S., Mathis C.A., Klunk W.E. (2001). Visualization of fibrillar amyloid deposits in living, transgenic *Caenorhabditis elegans* animals using the sensitive amyloid dye, X-34. Neurobiol. Aging.

[33] Regitz C., Dußling L.M., Wenzel U. (2014). Amyloid-beta (Aβ₁₋₄₂)-induced paralysis in *Caenorhabditis elegans* is inhibited by the polyphenol quercetin through activation of protein degradation pathways. Mol. Nutr. Food Res..

[34] Regitz C., Fitzenberger E., Mahn F., Dußling L., Wenzel U. (2015). Resveratrol reduces amyloid-beta (Aβ1–42)-induced paralysis through targeting proteostasis in an Alzheimer model of *Caenorhabditis elegans*. Eur. J. Nutr..

[35] Li J., Cui X., Ma X., Wang Z. (2017). rBTI reduced β-amyloid-induced toxicity by promoting autophagy-lysosomal degradation via DAF-16 in *Caenorhabditis elegans*. Exp. Gerontol..

[36] Neudegger T., Verghese J., Hayer-Hartl M., Hartl F.U., Bracher A. (2016). Structure of human heat-shock transcription factor 1 in complex with DNA. Nat. Struct. Mol. Biol..

[37] Drake J., Link C.D., Butterfield D.A. (2003). Oxidative stress precedes fibrillar deposition of Alzheimer’s disease amyloid beta-peptide (1–42) in a transgenic *Caenorhabditis elegans* model. Neurobiol. Aging.

[38] Hobert O. (2003). Behavioral plasticity in *C. elegans*: Paradigms, circuits, genes. J. Neurobiol..

[39] Kiew P.L., Don M.M. (2012). Jewel of the seabed: Sea cucumbers as nutritional and drug candidates. Int. J. Food Sci. Nutr..

[40] Lawson A.P., Klang J.A. (1993). Synthesis of 2-Tetrahydrofuranyl Ethers from Aqueous 4-Hydroxybutanal. Synth. Commun..

[41] Dahmen M., Marquardt W. (2016). Model-Based Design of Tailor-Made Biofuels. Energy Fuels.

[42] Tran L.-S., Wullenkord J., Li Y., Herbinet O., Zeng M., Qi F., Kohse-Höinghaus K., Battin-Leclerc F. (2019). Probing the low-temperature chemistry of di-n-butyl ether: Detection of previously unobserved intermediates. Combust. Flame.

[43] Diomede L., Rigacci S., Romeo M., Stefani M., Salmona M. (2013). Oleuropein aglycone protects transgenic *C. elegans* strains expressing Aβ42 by reducing plaque load and motor deficit. PLoS ONE.

[44] Wolff M., Zhang-Haagen B., Decker C., Barz B., Schneider M., Biehl R., Radulescu A., Strodel B., Willbold D., Nagel-Steger L. (2017). Aβ42 pentamers/hexamers are the smallest detectable oligomers in solution. Sci. Rep..

[45] Pryor N.E., Moss M.A., Hestekin C.N. (2012). Unraveling the early events of amyloid-β protein (Aβ) aggregation: Techniques for the determination of Aβ aggregate size. Int. J. Mol. Sci..

[46] Karamanos T.K., Kalverda A.P., Thompson G.S., Radford S.E. (2015). Mechanisms of amyloid formation revealed by solution NMR. Prog. Nucl. Magn. Reson. Spectrosc..

[47] Garcia-Osta A., Alberini C.M. (2009). Amyloid beta mediates memory formation. Learn Mem..

[48] Puzzo D., Arancio O. (2013). Amyloid-β peptide: Dr. Jekyll or Mr. Hyde?. J. Alzheimers Dis..

[49] Brothers H.M., Gosztyla M.L., Robinson S.R. (2018). The Physiological Roles of Amyloid-β Peptide Hint at New Ways to Treat Alzheimer’s Disease. Front. Aging Neurosci..

[50] Urbanc B., Cruz L., Yun S., Buldyrev S.V., Bitan G., Teplow D.B., Stanley H.E. (2004). In silico study of amyloid beta-protein folding and oligomerization. Proc. Natl. Acad. Sci. USA.

[51] Salminen A., Ojala J., Suuronen T., Kaarniranta K., Kauppinen A. (2008). Amyloid-beta oligomers set fire to inflammasomes and induce Alzheimer’s pathology. J. Cell Mol. Med..

[52] Larson M.E., Lesné S.E. (2012). Soluble Aβ oligomer production and toxicity. J. Neurochem..

[53] Yi X., Jiang S., Qin M., Liu K., Cao P., Chen S., Deng J., Gao C. (2020). Compounds from the fruits of mangrove Sonneratia apetala: Isolation, molecular docking and antiaging effects using a *Caenorhabditis elegans* model. Bioorg. Chem..

[54] Kumsta C., Chang J.T., Schmalz J., Hansen M. (2017). Hormetic heat stress and HSF-1 induce autophagy to improve survival and proteostasis in *C. elegans*. Nat. Commun..

[55] Sultana R., Perluigi M., Butterfield D.A. (2009). Oxidatively modified proteins in Alzheimer’s disease (AD), mild cognitive impairment and animal models of AD: Role of Abeta in pathogenesis. Acta Neuropathol..

[56] Butterfield D.A. (1997). beta-Amyloid-associated free radical oxidative stress and neurotoxicity: Implications for Alzheimer’s disease. Chem. Res. Toxicol..

[57] Cheignon C., Tomas M., Bonnefont-Rousselot D., Faller P., Hureau C., Collin F. (2018). Oxidative stress and the amyloid beta peptide in Alzheimer’s disease. Redox Biol..

[58] Kienlen-Campard P., Miolet S., Tasiaux B., Octave J.N. (2002). Intracellular amyloid-beta 1–42, but not extracellular soluble amyloid-beta peptides, induces neuronal apoptosis. J. Biol. Chem..

[59] Umeda T., Ramser E.M., Yamashita M., Nakajima K., Mori H., Silverman M.A., Tomiyama T. (2015). Intracellular amyloid β oligomers impair organelle transport and induce dendritic spine loss in primary neurons. Acta Neuropathol. Commun..

[60] Chokchaisiri R., Chaneiam N., Svasti S., Fucharoen S., Vadolas J., Suksamrarn A. (2010). Labdane Diterpenes from the Aerial Parts of *Curcuma comosa* Enhance Fetal Hemoglobin Production in an Erythroid Cell Line. J. Nat. Prod..

[61] O’Mathúna D.P., Doskotch R.W. (1995). New labdane diterpene glycosides from *Amphiachyris amoena*. J. Nat. Prod..

[62] Di Pietro M.E., Mannu A., Mele A. (2020). NMR Determination of Free Fatty Acids in Vegetable Oils. Processes.

[63] Namikoshi M., Fujiwara T., Nishikawa T., Ukai K. (2006). Natural Abundance (14)C Content of Dibutyl Phthalate (DBP) from Three Marine Algae. Mar. Drugs.

[64] Rohn I., Raschke S., Aschner M., Tuck S., Kuehnelt D., Kipp A., Schwerdtle T., Bornhorst J. (2019). Treatment of *Caenorhabditis Elegans* with Small Selenium Species Enhances Antioxidant Defense Systems. Mol. Nutr. Food Res..

[65] Smith J.V., Luo Y. (2003). Elevation of oxidative free radicals in Alzheimer’s disease models can be attenuated by Ginkgo biloba extract EGb 761. J. Alzheimers Dis..

[66] Margie O., Palmer C., Chin-Sang I. (2013). *C. elegans* chemotaxis assay. J. Vis. Exp..

