# Supplementary material for: Beneficial Effects of Cyclic Ether 2-Butoxytetrahydrofuran from Sea Cucumber Holothuria scabra against Aβ Aggregate Toxicity in Transgenic Caenorhabditis elegans and Potential Chemical Interaction
Source: Molecules. 2021 Apr 11;26(8):2195. doi: 10.3390/molecules26082195 (PMC8070609; doi:10.3390/molecules26082195)
Supplement: Supplementary file 1 [file molecules-26-02195-s001.pdf]

Supplementary Materials

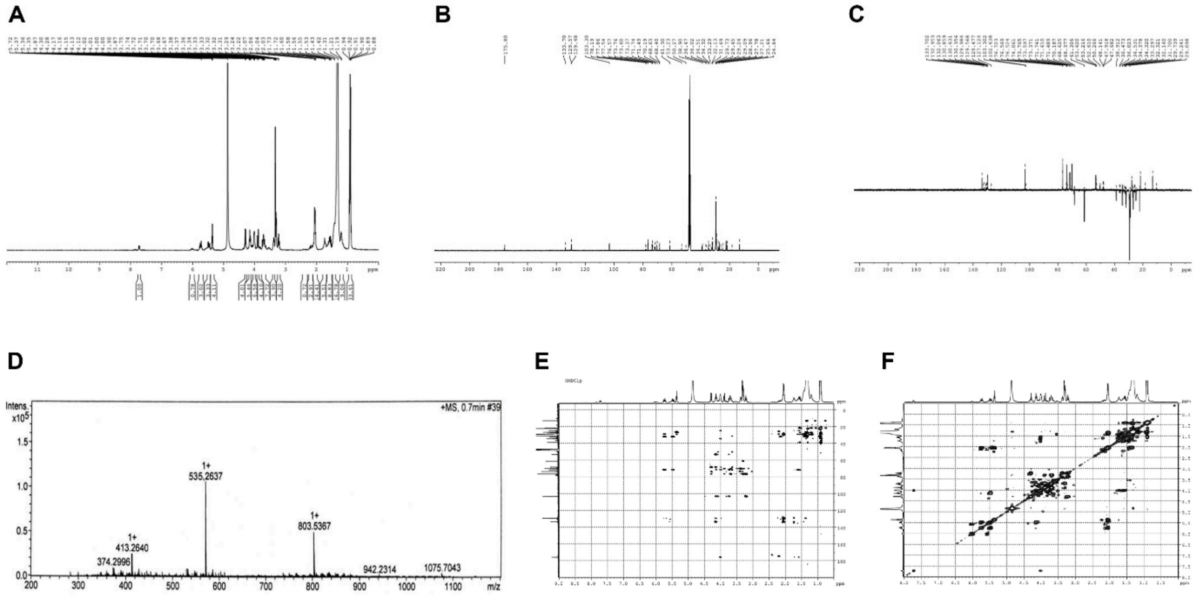

Figure S1. Chemical analyses of Compound **1** (Holothuria A). Diagrams illustrate ^{1}H NMR (A), ^{13}C NMR (B), DEPT 135 (C), HMBC (E), and COSY (F) spectra in CD_3OD. Formula as $C_{26}H_{40}O_{10}$ with Mol. Wt. = 512, HR-TOFMS (ES+) m/z 535.2637 $[M+Na]^+$, calcd for $C_{26}H_{40}O_{10}$+Na (D).

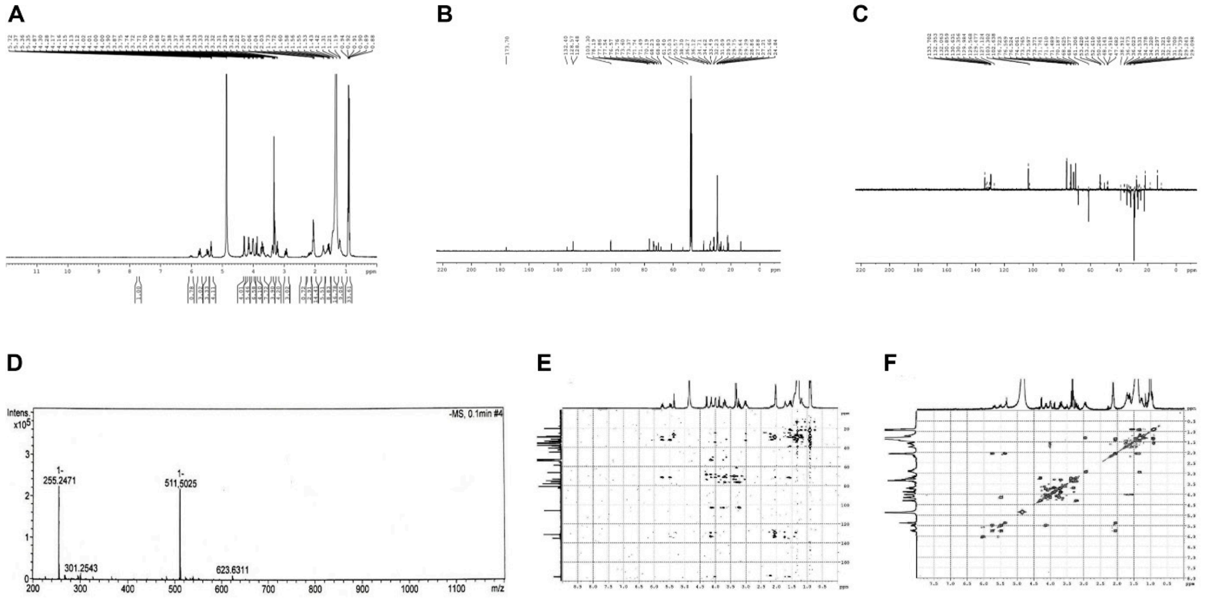

Figure S2. Chemical analyses of Compound **2** (Holothuria B). Diagrams illustrate ^{1}H NMR (A), ^{13}C NMR (B), DEPT 135 (C), HMBC (E), and COSY (F) spectra in CD_3OD. Formula as $C_{26}H_{40}O_{10}$ with Mol. Wt. = 512, HR-TOFMS (ES+) m/z 511.5025 $[M-H]^-$, calcd for $C_{26}H_{40}O_{10}$-H (D).

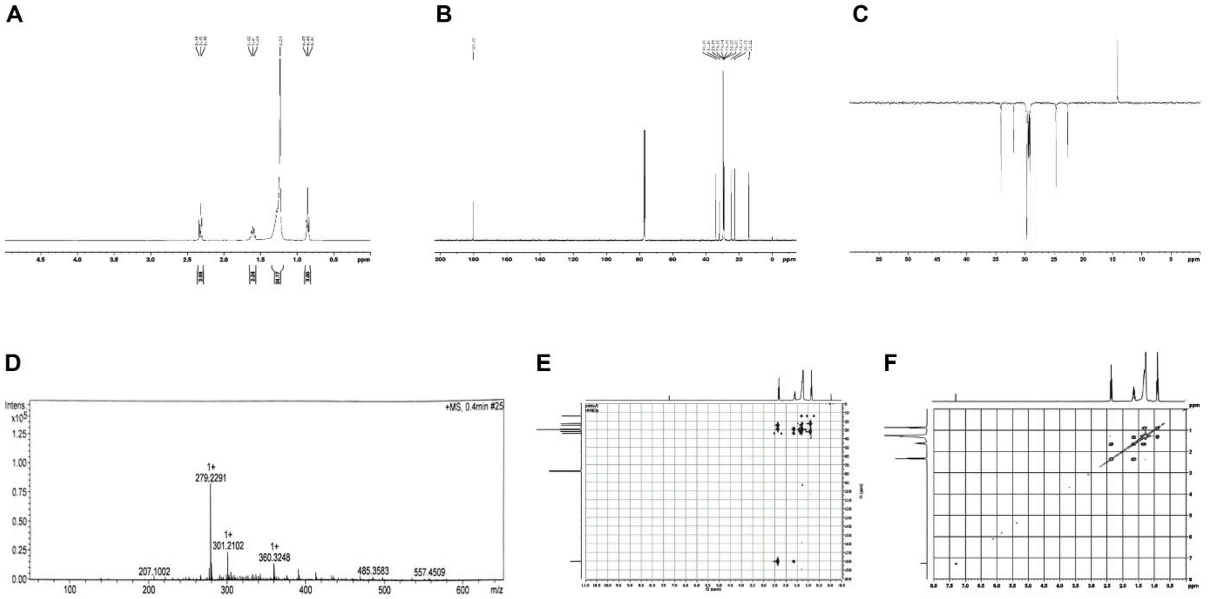

Figure S3. Chemical analyses of Compound **3** (Palmitic acid). Diagrams illustrate ^{1}H NMR (A), ^{13}C NMR (B), DEPT 135 (C), HMBC (E), and COSY (F) spectra in $CDCl_3$. Formula as $C_{16}H_{32}O_2$ with Mol. Wt. = 256, HR-TOFMS (ES+) m/z 279.2291 $[M+Na]^+$, calcd for $C_{16}H_{32}O_2$+Na (D).

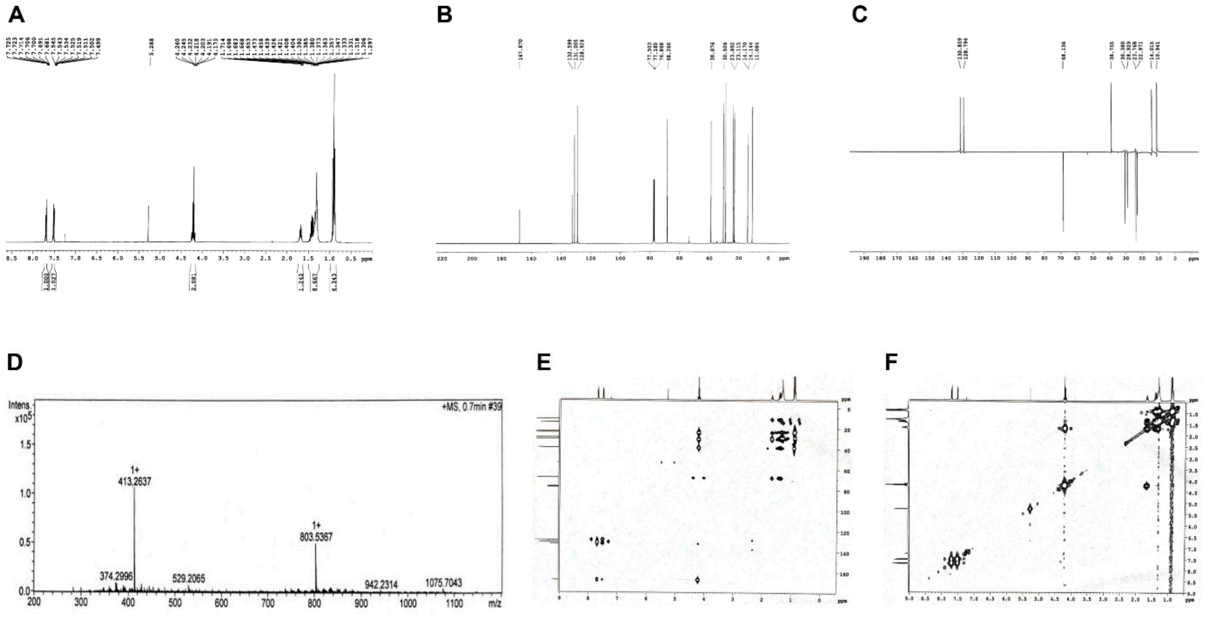

Figure S4. Chemical analyses of Compound **4** (Bis (2-ethylhexyl) phthalate). Diagrams illustrate ^{1}H NMR (A), ^{13}C NMR (B), DEPT 135 (C), HMBC (E), and COSY (F) spectra in $CDCl_3$. Formula as $C_{24}H_{38}O_4$ with Mol. Wt. = 390, HR-TOFMS (ES+) m/z 413.2682 $[M+Na]^+$, calcd for $C_{24}H_{38}O_4$+Na (D).

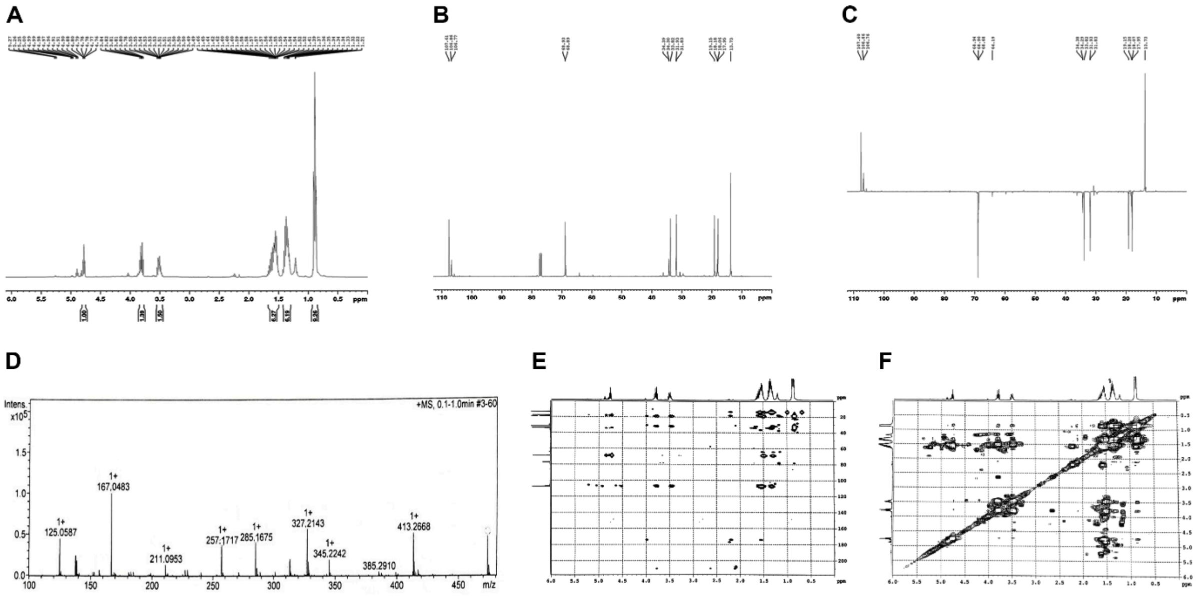

Figure S5. Chemical analyses of Compound **5** (2-BTHF). Diagrams illustrate ^{1}H NMR (A), ^{13}C NMR (B), DEPT 135 (C), HMBC (E), and COSY (F) spectra in $CDCl_3$. Formula as $C_8H_{16}O_2$ with Mol. Wt. = 144, HR-TOFMS (ES+) m/z 167.3124 $[M+Na]^+$, calcd for $C_8H_{16}O_2$+Na (D).

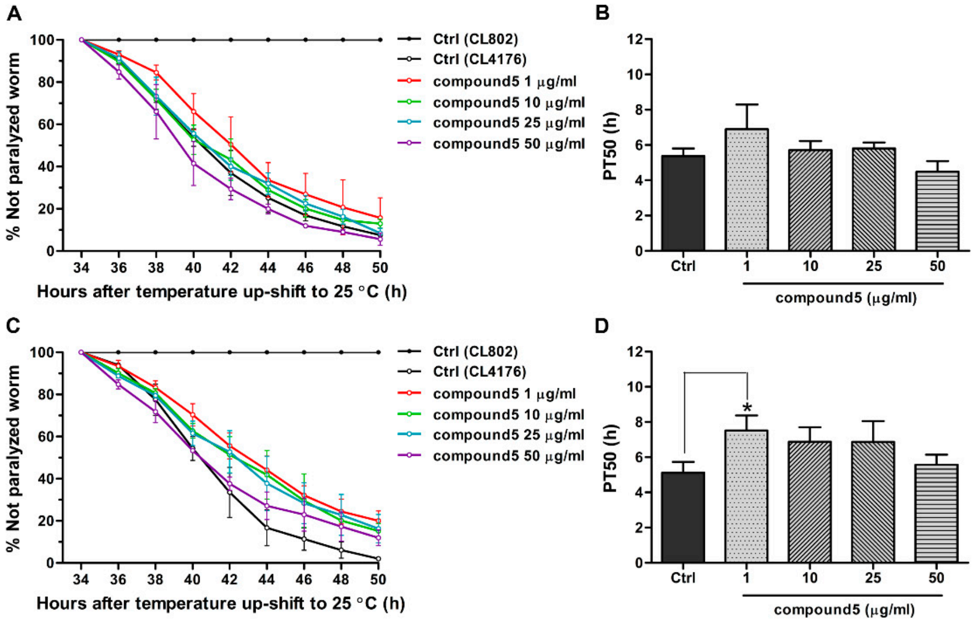

Figure S6. Effects of Compound **5** (2-BTHF) on Aβ-induced paralysis of *C. elegans* strain CL4176 in treatment before or after temperature up-shifting. Time course of Aβ-induced paralysis in transgenic *C. elegans* strain CL4176 treated with or without Compound **5** (1-50 μg/ml) before (A) or after (C) Aβ induction by temperature up-shifting and CL802 control strain. The paralysis was scored at 2 h intervals. Data are expressed as percentages ± SD of unparalyzed worms from three independent assays with at least 100 worms in each experiment. For quantitative analysis, PT_{50} values (mean time duration at which 50% worms were paralyzed) were calculated from paralysis curves obtained from Compound **5** treatment before (B) or after (D) Aβ induction. Error bars indicate SD. $^{*}p < 0.05$ vs. untreated control CL4176. The data indicated that anti-paralytic effect mediated by Compound **5** in all treatment was declined when the worms were subjected to treatment before or after the temperature increase, respectively

Table S1. ^{1}H-, ^{13}C-NMR and HMBC data of Compound **1** (Holothuria A) in CD_3OD

Positions	^{1}H-NMR	^{13}C-NMR	HMBC
1	1.20 (m)	38.9	2, 19, 11
2	1.43 (m)	24.8	3
3	1.31 (m)	22.3	4
4	2.06 (m)	31.6	5
5	-	32.1	-
6	4.01 (m)	71.7	7
7	5.49 (dd, 8.0, 16.0 Hz)	129.5	6
8	5.74 (dd, 8.0, 16.0 Hz)	133.7	6, 7
9	1.53 (m)	27.7	19, 1, 10
10	-	27.7	-
11	2.04 (m)	27.2	12
12	5.37 (dd, 8.0, 4.0 Hz)	129.4	11, 13
13	-	132.9	-
14	1.58, 1.72 (m)	34.5	12, C=O
15	-	175.8	-
16	4.16 (m)	71.4	17, C=O
17	4.00 (m)	53.2	18
18	3.74, 4.13 (m)	68.4	16, 17
19	0.91	13.1	1, 11
20	0.89	21.7	4, 3
1'	4.29 (1H, d, 8.0 Hz)	103.3	18, 2', 3', 4'
2'	3.22 (1H, t, 8.0 Hz)	73.6	3'
3'	3.37 (1H, dd, 8.0, 4.0 Hz)	77.5	2', 5'
4'	3.32 (1H, t, 8.0 Hz)	77.8	3'
5'	3.29 (1H, m)	70.1	6'
6'	3.68 (1H, dd, 12.0, 4.0 Hz)	61.3	3', 4'
	3.89 (1H, dd, 12.0, 4.0 Hz)		

Table S2. ^{1}H-, ^{13}C-NMR and HMBC data of Compound **2** (Holothuria B) in CD_3OD

Positions	^{1}H-NMR	^{13}C-NMR	HMBC
1	1.18 (m)	22.3	2
2	1.51 (m)	27.8	3, 4
3	2.89 (m)	71.4	4
4	2.19 (m)	38.8	3
5	-	32.1	-
6	1.49, 1.53 (m)	34.4	7
7	5.40 (dd, 8.0, 16.0 Hz)	128.5	6
8	5.65 (dd, 8.0, 16.0 Hz)	132.4	6, 7
9	1.53 (m)	27.8	19, 1, 10
10	-	50.2	-
11	2.19 (m)	27.2	12
12	5.28 (dd, 8.0, 4.0 Hz)	128.4	11, 13
13	-	132.1	-
14	1.53, 1.51 (m)	34.7	12, 13, C=O
15	-	173.7	-
16	4.16 (m)	71.7	17, C=O
17	4.05 (m)	53.0	18
18	3.66, 4.15 (m)	68.4	16, 17
19	0.86	13.1	1, 11, 10
20	0.85	21.7	4, 3, 5
1'	4.27 (1H, d, 8.0 Hz)	103.3	17, 2', 3', 4'
2'	3.15 (1H, m)	73.6	3'
3'	3.25 (1H, m)	76.5	2', 5'
4'	3.16 (1H, m)	76.5	3'
5'	3.15 (1H, m)	70.1	6'
6'	3.82, 3.62 (1H, d, 12.0 Hz)	61.4	3', 4'

Table S3. ^{1}H-, ^{13}C-NMR and HMBC data of Compound **3** (Palmitic acid) in $CDCl_3$

Positions	^{1}H-NMR	^{13}C-NMR	HMBC
COOH	-	178.4	-
2	2.32 (t, 7.6 Hz)	34.0	1, 3
3	1.61 (m)	24.7	2, 4
4	1.23 (m)	29.0	3, 5
5	1.23 (m)	29.3	4, 6
6-12	1.23 (m)	29.6	13
13	1.23 (m)	29.3	12, 14
14	1.23 (m)	31.9	13, 15
15	1.23 (m)	22.7	14, 16
16	0.86 (t, 7.2 Hz)	14.1	15

Table S4. ^{1}H-, ^{13}C-NMR and HMBC data of Compound **4** (Bis (2-ethylhexyl) phthalate) in $CDCl_3$

Positions	^{1}H-NMR	^{13}C-NMR	HMBC
1, 1'	0.91 (t,)	14.1	2
2, 2'	1.29 (m)	23.1	1, 3
3, 3'	1.31 (m)	29.1	2, 4
4, 4'	1.35 (m)	30.5	3, 5
5, 5'	1.68 (m)	38.8	4, 6, 8
6, 6'	4.21 (m)	68.2	5, 7
7, 7'	-	167.8	-
8, 8'	1.42 (m)	23.8	5, 9
9, 9'	0.93 (t,)	11.0	8
10, 10'	-	132.5, 132.6	-
11, 11'	7.70 (dd, 6.0, 3.2 Hz)	131.0, 128.9	10, 10'
12, 12'	7.52 (dd, 6.0, 3.2 Hz)	132.5	

Table S5. ^{1}H-, ^{13}C-NMR and HMBC data of Compound **5** (2-BTHF) in $CDCl_3$

Positions	^{1}H-NMR	^{13}C-NMR	HMBC
1	-	-	-
2	3.78 (m)	68.6	3, 5
3	1.5 (m)	31.9	2, 4
4	1.38 (m)	19.1	3, 5
5	4.75 (m)	107.6	2, 7
6	-	-	-
7	3.50 (m)	68.9	5, 8, 9
8	1.60 (m)	34.2	7, 9
9	1.30 (m)	18.1	8, 10
10	0.87 (t, 8.0 Hz)	13.7	8, 9
